# Comparing the Performance of NoSQL Approaches for Managing Archetype-Based Electronic Health Record Data

**DOI:** 10.1371/journal.pone.0150069

**Published:** 2016-03-09

**Authors:** Sergio Miranda Freire, Douglas Teodoro, Fang Wei-Kleiner, Erik Sundvall, Daniel Karlsson, Patrick Lambrix

**Affiliations:** 1 Departamento de Tecnologia da Informação e Educação em Saúde, Universidade do Estado do Rio de Janeiro, Rio de Janeiro, RJ, Brazil; 2 Department of Biomedical Engineering, Linköping University, Linköping, Sweden; 3 Department of Computer and Information Science, Linköping University, Linköping, Sweden; 4 Region Östergötland, Linköping, Sweden; 5 Swedish e-Science Research Centre, Linköping, Sweden; University of Western Australia, AUSTRALIA

## Abstract

This study provides an experimental performance evaluation on population-based queries of NoSQL databases storing archetype-based Electronic Health Record (EHR) data. There are few published studies regarding the performance of persistence mechanisms for systems that use multilevel modelling approaches, especially when the focus is on population-based queries. A healthcare dataset with 4.2 million records stored in a relational database (MySQL) was used to generate XML and JSON documents based on the openEHR reference model. Six datasets with different sizes were created from these documents and imported into three single machine XML databases (BaseX, eXistdb and Berkeley DB XML) and into a distributed NoSQL database system based on the MapReduce approach, Couchbase, deployed in different cluster configurations of 1, 2, 4, 8 and 12 machines. Population-based queries were submitted to those databases and to the original relational database. Database size and query response times are presented. The XML databases were considerably slower and required much more space than Couchbase. Overall, Couchbase had better response times than MySQL, especially for larger datasets. However, Couchbase requires indexing for each differently formulated query and the indexing time increases with the size of the datasets. The performances of the clusters with 2, 4, 8 and 12 nodes were not better than the single node cluster in relation to the query response time, but the indexing time was reduced proportionally to the number of nodes. The tested XML databases had acceptable performance for openEHR-based data in some querying use cases and small datasets, but were generally much slower than Couchbase. Couchbase also outperformed the response times of the relational database, but required more disk space and had a much longer indexing time. Systems like Couchbase are thus interesting research targets for scalable storage and querying of archetype-based EHR data when population-based use cases are of interest.

## Introduction

In traditional Electronic Health Record (EHR) systems, data are usually persisted in relational or object-relational databases. These systems fall usually into the Online Transactional Processing (OLTP) category, using the Entity/Relationship (ER) model mapped into database tables. They are used in general to store and retrieve individual patient records for healthcare purposes. In this scenario, the queries are issued using key parameters, such as EHR and patient identifiers, which are trivially indexed.

EHR data can also be used for other purposes than the direct care, such as healthcare quality and performance assessment, clinical and epidemiological studies, determination of a population epidemiological profile, service management, etc. In these situations, we do not only search data for a specific known patient, but for a subset of patients that meet certain searching criteria. We call this kind of queries population-based queries. Although transactional systems may, and often are, used for providing answers to such queries, the system performance may suffer depending on the complexity of and the number of queries submitted to the database. In such cases, and especially when aiming for data analysis, it is common to transform and load data into a storage structure more optimized for population-based queries, a data warehouse (DW), in order to separate responsibilities and keep the performance at an acceptable level. In a DW environment, data are organized and maintained through the use of dimensional modelling [1,2]. Such systems usually fall into the category of Online Analytical Processing (OLAP). Both traditional OLTP and OLAP systems are resource demanding to maintain in a healthcare environment where changes and new requirements frequently appear.

Generic healthcare data modelling [3–8] has been proposed as a flexible alternative to the traditional data models, since they do not require changing the model as new data requirements arise. However, this flexibility raises the issue of how to manage data expressed in such models in healthcare databases. This paper evaluates some NoSQL approaches for storing and retrieving clinical data generated according to the openEHR multilevel model in terms of storage space and response time for population-based queries. The manuscript is organized as follows. The background section gives an overview of recent multilevel models for clinical data, focusing on openEHR and some proposals for its data persistence. The methods section describes the databases, the datasets and the queries used in the evaluation. The results of the evaluation are shown in the results section followed by the discussion and limitations of the study.

## Background

**Fig 1:**
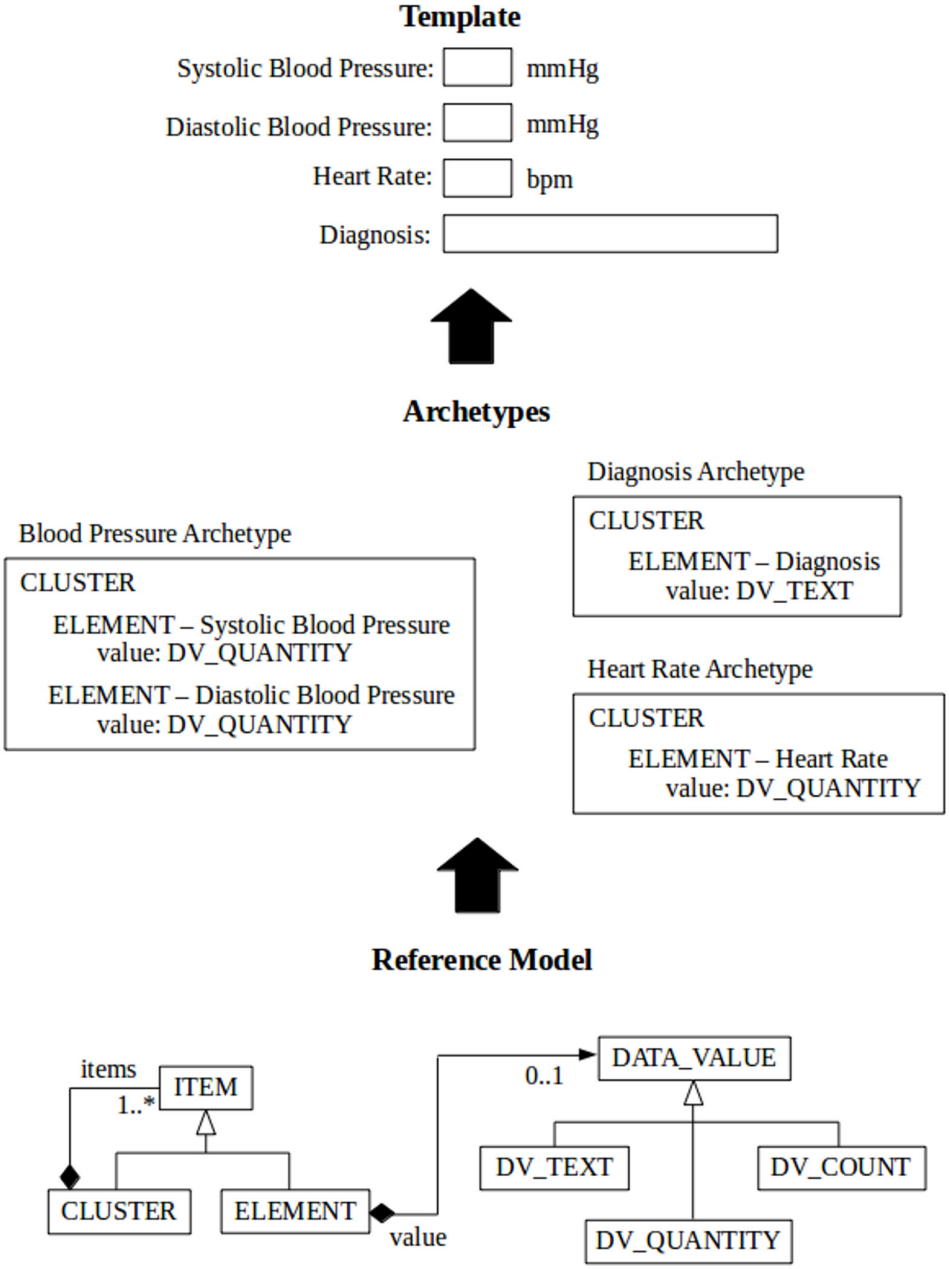
A simplified view of openEHR multilevel model. Archetypes are created from the building blocks of the RM to express domain concepts which represent valid data structure in the RM. By combining archetypes we can generate templates that may be used to generate forms or messages.

Several multilevel approaches to model healthcare data have been proposed in the past decade: openEHR [5], ISO 13606 standard [6], HL7-CDA (Clinical Document Architecture) [7], and the more recent Clinical Information Modelling Initiative (CIMI) [8]. ISO 13606 and HL7-CDA are concerned with the communication of extracts of EHRs between EHR systems. CIMI is an international collaboration dedicated to providing a common format for detailed specifications for the representation of health information content. The openEHR specifications aim at providing a way of implementing more flexible EHRs, by gracefully embracing change. The openEHR approach represents healthcare information using multiple levels of models (Fig 1). At the bottom there is a stable general *reference model* (RM) that contains commonly occurring structures/patterns that provide “building blocks” used by the upper, more use-case specific (and thus more frequently changing) levels. In the RM, classes like EHR, COMPOSITION, ENTRY, CLUSTER and ELEMENT, for instance, form a hierarchy of generic components of an EHR model.

The levels above the RM in openEHR contain models of domain concepts, called *archetypes* and *templates*, which are expressed as constraints on structures made out of the reference model building blocks [5], using an archetype model (AM). An archetype can be explained as a meta-model describing the names and meanings of the RM pieces used for a particular structure that models the documentation of a scope-limited clinical concept or set of concepts. For instance, an archetype may specify how ELEMENTs may be combined in an ENTRY in order to represent the concept “body mass index”, or how different ENTRY instances may be combined in order to make a paediatric consultation (COMPOSITION), and so on.

The openEHR specifications aim at accommodating new requirements primarily at the AM (archetype and template) level without the need to change the RM and its associated storage mechanism, thus requiring fewer changes in the application code and persistence mechanism [9]. This approach also promotes semantic interoperability between systems based on the shared RM, configured using (partly) shared archetypes and templates, but the interoperability aspect is not the focus of this paper.

The exact storage and retrieval of openEHR-based information is not dictated by the specifications, instead it is an open issue where different approaches are being used and researched. The issue of database performance is one that occasionally emerges in the openEHR technical discussion list [10, 11] and in tutorials about the subject. Archetype-based systems should ideally show good performance for single-patient queries, that is, when we are interested in getting data from an EHR of a single patient, and also for OLAP analysis. In addition they should allow population-based ad hoc queries, that is, queries that are not planned in advance, thus having no query-specific indexing optimizations, or whose results could not be easily retrieved from a data warehouse.

Arguably the most used persistence mechanism in object-oriented systems is the Object-Relational Mapping (ORM) which adapts the classes in the object model to the tables in the relational model. Due to the relative deep hierarchy and complexity of the openEHR archetypes and reference model, a number of joins would be necessary just to retrieve small pieces of patient data from the database. Therefore, one may put into question the efficiency of the ORM approach for openEHR-based systems. In fact, this is Muñoz et al [12] conclusion when experimenting with a ORM in a prototype of a system based on the ISO 13606 standard, which has a similar approach to openEHR, but with a simpler reference model.

A survey of openEHR-based systems worldwide [13] found a number of implementations, most of them experimental, with a variety of approaches to store data. Usually they are built on top of relational database management systems (relational DBMS) with some workarounds to deal with the impedance mismatch between the relational model and the logically tree-structured archetype-based EHR. This may be explained by the widespread experience with relational DBMS and several proposals based on the openEHR wiki (Node + path persistence) [14], which basically use the relational model as a support for storing BLOBs indexed by paths defined by the archetypes. In a recent study not included in that survey, Wang et al [15], instead of using the ORM or the BLOB approaches, mapped the archetypes (not the reference model) to the relational model. Their experiment compared a traditional EHR system based on the relational model to the archetype-relational mapping and to the BLOB approach. The archetype-relational mapping showed similar performance to the relational model for the single-patient queries and were much more efficient for the population-based queries. The BLOB approach required much more time than the other approaches for both types of queries. To our knowledge this is the first study that questions the efficiency of the Node + path persistence approach.

In the Frade et al survey [13], the authors found that some systems have adopted an XML database as the storage backend. XML is an interesting solution for openEHR persistence mechanisms due to its flexibility but also, and more importantly, the existence of fairly mature openEHR XML specifications and tools [16] that can serialize openEHR objects into XML format. These motivations are behind our focus on exploring XML databases in the LiU-EEE openEHR server [17] and in a comparison of XML databases [18], which tested the performance of four XML databases (BaseX, eXistdb, Sedna and Berkeley DB XML) without any query specific optimizations. The motivation behind this study was the assumption that database indexing are unlikely to be optimized for ad hoc population-based queries. The results showed that while XML databases, configured with default indexes, can answer single-patient queries in the order of milliseconds, they do not scale up gracefully to provide satisfactory response time when ad hoc population-based queries are submitted to larger datasets. Similar conclusions were reached by Green [19], who compared the response times of two XML databases (Sedna and eXistdb) with the MySQL relational database in a project which dealt with resources management in advanced computer installations. Another study [20] compared three database approaches (eXistdb, XML enabled database in Microsoft SQL Server–MS-SQL, and a generalized key-value pair table in MS-SQL) for storing HL7-CDA documents. The authors measured the response times to five population-based queries of different complexity and the results showed that the XML database was much slower than the other two approaches.

Marand [21], an openEHR vendor, uses an inverted index implemented with Apache Lucene [22] to handle their queries. Marco-Ruiz et al [23] built an openEHR-based repository, using the Marand’s system, to convert, transform and aggregate patient legacy data from laboratory systems in Norway. With data of about 230,000 patients, its performance was evaluated with some populational-based queries, using indicators for pertussis and salmonela. The response times varied from 0.656 to 2.419s in a computer with an Intel Xenon 2.9 GHz processor, 12 GB RAM. The authors claimed that this performance should be improved because this system is to be used by clinicians.

Another flexible openEHR-compatible format (e.g., via direct XML conversion) is JSON, for which there are several NoSQL JSON-based databases [24, 25]. NoSQL databases are a heterogeneous family of products, including XML databases, that claim to deal in a more natural way with unstructured or semi-structured data than the relational databases. Kimura and Ishihara [26] implemented a virtual file system, having MongoDB as the NoSQL backend, for processing a high volume of HL7 messages with high scalability. Outside the healthcare arena, Parker et al [27] compared the performance of MongoDB [25], a JSON-based NoSQL database, with Microsoft SQL Server, using a modest-sized structured database. MongoDB had better performance for insertions, updates and simple queries, while SQL Server was faster with queries involving attributes that did not belong to a key and with aggregate queries (population-based queries)

As shown above, several persistence mechanisms for openEHR have been tested, as new groups and companies start implementing openEHR-based systems. To the best of our knowledge, there is no study in the literature that deals with the persistence of openEHR records in any JSON NoSQL backend. This paper further investigated the performance on population-based queries of NoSQL database management systems storing openEHR-based data. We expanded the scope of [18], covering also a state-of-the-art MapReduce backend with a unique indexing mechanism. We also tried to optimize the retrieval performance of the XML databases through query rewriting and/or attribute indexing.

## Materials and Methods

To simulate population-based data management in real healthcare research environments, this study required a data source with millions of archetype-based health records and a set of accompanying realistic epidemiology queries. To simulate such environment, we engineered a solution that created a large openEHR dataset from existing relational healthcare databases containing administrative and epidemiological data. Furthermore, we created equivalent epidemiological queries for each of the database solutions.

### Test Data

This study generated openEHR-based documents using data from four relational databases:

1. The hospitalizations database (AIH), of the Brazilian Public Hospital Information System for the State of Rio de Janeiro, from January 2000 to June 2004. AIH contains data about demographics, admission, procedures, diagnosis and death.
2. The oncology module of the high complexity procedures (outpatient) database (APAC) of the Brazilian Public Ambulatory Information System for the State of Rio de Janeiro, from January 2000 to June 2004, containing demographic, administrative, treatment and diagnosis data.
3. Mortality Information System (MIS) for the state of Rio de Janeiro from January 2000 to June 2004, with data about the death of individuals.
4. The database of the National Cervical Cancer Information System–SISCOLO–for the State of Rio de Janeiro, Brazil, from June 2006 to December 2009. This database contains two tables (histology and cytology) with data about cervical pathological examinations, Pap smear tests, in addition to demographics data.

These databases were subjected to a record linkage process, so that records belonging to the same patient received the same identification number (uid). This process was performed separately for AIH, APAC and MIS [28], on one side, and for the SISCOLO database on the other side [29].

After the linkage process, these databases were imported into a relational schema of MySQL [30]. The histology table has 7,477 records belonging to 6,238 patients. The cytology table has 2,471,088 records belonging to 1,679,801 patients, with 5,316 of them having records in both tables. The outpatient table has 559,698 records belonging to 53,435 patients, where 6,931 patients have just one record, 9,267 have two records and 14,943 have more than 10 records. A total of 33,636 patients also have records in the hospitalization table and 22,024 have a record in the mortality database. Finally, the AIH database has 4,360,912 records belonging to 2,541,978 patients, where 1,864,029 patients have only one hospitalization, 401,118 have two hospitalizations and 27,233 have more than 10 hospitalizations.

### Generating openEHR Record Documents

In openEHR-based systems data is committed in logically-versioned chunks called *compositions*. For the SISCOLO database, a set of 11 archetypes and 3 templates was designed from scratch using the Ocean Informatics Archetype Editor [31] and the Template Designer [32]. To represent the contents of the SISCOLO schema, each template corresponds to a type of composition: one composition with the demographic data of the women (date of birth and educational level) and two event compositions corresponding to the histologic and cytologic examinations. For the APAC and AIH databases, 8 archetypes were either created from scratch or reused from the online openEHR clinical knowledge manager (CKM) [33] and 2 templates were designed for each database: a template for the demographic data and another template containing the event composition for the outpatient and hospitalization claims, respectively.

The content of the relational database was mapped to openEHR documents according to the following steps:

1. XML files (EHR data instance examples) corresponding to each of the templates were generated using LiU-EEE [17].
2. These XML documents were then used as a basis for a manually-edited Freemarker [34] template. Using this template, a sample of records in the relational database were mapped to openEHR compositions. All compositions from a patient were put into a dedicated directory.
3. For each directory of compositions created in step 2, an XML document representing the whole patient record (or EHR) with all its compositions was then generated through LiU-EEE software [17].
4. Similar to step 2, the XML documents generated by LiU-EEE were manually edited into another Freemarker template. Then, this template was used to map all records in the databases to openEHR XML documents. One XML document was created for each patient, containing all data belonging to each patient.
5. Finally, a Freemarker template for JSON was also generated from the XML Freemarker template. The JSON template was used to map all records in the databases to openEHR JSON documents.

The XML and JSON documents generated in the process above were then stored into different kinds of storage solutions as described in the following section.

### Evaluated Storage Systems

This study evaluated the following storage solutions: BaseX, version 7.6 [35], eXistdb, version 2.1 [36], Berkeley DB XML, version 2.5.16 [37], and Couchbase, version 2.0.1 [38]. The MySQLdatabase, version 5.5.46, was used as a baseline benchmark.

The XML databases using their default indexes were already assessed in a previous paper [18]. This time, however, query rewriting and customized indexing were performed specifically for each database in an attempt to optimize the retrieval performance. This is explained in the sections “query rewriting” and “indexing” below.

Couchbase was designed especially for distributed computing and has native support for JSON documents. The database is queried by means of MapReduce procedures [39] where in the map phase, filtering and sorting operations are applied to the data, and in the reduce phase, the mapped data are summarized. Query support includes specific key lookups, range queries, and aggregate lookups. Although XML documents can be stored in Couchbase, it does not parse XML server side as it does with JSON. There is an extension for parsing XML that is not part of the engine that Couchbase uses to process the javascript map functions [40]. The indexes, called Views in the Couchbase documentation, are created for each query. Views can be parameterized so that it could be queried for different values of its parameters without the need of reindexing. Indexing is evenly distributed across all nodes in a cluster, with each node indexing the data it holds. Indexes are updated incrementally when the data is added or changed. Incremental MapReduce provides powerful aggregates for real-time analytics, even with large datasets.

In summary the backends compared are XML databases (BaseX, eXistdb and Berkeley DB XML) and a MapReduce backend with native support to JSON (Couchbase), taking an original relational database as a reference (MySQL).

### Evaluation Setup

The evaluation was done in terms of storage space and response time to a series of queries against each of the storage proposals presented in the previous section. Six datasets of the original SUS databases were generated, each consisting of a stratified random sample from the histology, cytology, APAC and AIH tables, where the number of patient records belonging to each of the four tables is proportional to the number of patients in each table. The number of patients in each dataset is:

1. sus10k—10,000;
2. sus42k—42,428;
3. sus100k—100,000;
4. sus420k—424,270;
5. sus1000k—1,000,000;
6. sus4200k—4,242,500.

The XML files of each dataset were stored in the XML databases and the corresponding JSON files were stored in Couchbase.

A set of eight population-based queries was defined and written according to each database query language:

Q1) What are the EHRs with a null-flavour date of Papanicolaou exam within the period X?
Q2) What are the EHRs with a percentage of rejected sample of cytological exams for any reason in the period X?
Q3) What are the EHRs with histologic exams in the period X?
Q4) What are the EHRs with the diagnostic “C50” (Malignant neoplasm of breast) in the period X in the outpatient data?
Q5) What are the EHRs with the diagnostic “F205” (Residual schizophrenia) in the period X in the hospitalization data?
Q6) What are the EHRs of patients with metastasis and with diagnoses “C50” in the period X?
Q7) What are the EHRs of patients with diagnosis “C50” born in the period X in the outpatient data?
Q8) What are the EHRs of patients with diagnosis “D069” (Carcinoma in situ of cervix, unspecified) born in the period X in the hospitalization data?

Three of these queries came from an epidemiological study that evaluated the effectiveness of the SISCOLO screening programme [41] and the other five were created to represent the general epidemiological profile of the population. They do not constitute an exhaustive list of possible queries, but were framed in order to show different levels of complexity, such as presence of a specific element within a second-level archetype and a specific attribute within this element (Q1); four disjunctive clauses within a second-level archetype (Q2); search in only a high level archetype (Q3); attribute equality within an element of a second-level archetype that are unique for each patient (Q4); attribute equality within an element of a second-level archetype that are non-unique for each patient (Q5); two conjunctive clauses within a second-level archetype (Q6); attribute equality within an element of a second-level archetype (Q7); simultaneous search in two high-level archetypes and in two conjunctive clauses within one second-level and one third-level archetypes (Q8).

The queries were framed to return all record identifiers that satisfied the searching criteria. Therefore, only map routines in Couchbase were codified. A key searching criterion in all the queries was the date interval. Shorter and longer periods were specified for each query to evaluate the performance for different result set sizes.

### Query Rewriting

The XML queries written originally using full paths in the XQuery query language [42] were rewritten in different ways in order to verify which has the best performance in the XML databases. The changes made in the queries were:

1. retrieving the owner_id/id/value attribute of the VERSIONED-OBJECT class in the openEHR Reference Model instead of the ehr_id/id/value attribute of the EHR class. This means that the retrieved attribute should be searched for in a lower level of the XML document hierarchy.
2. replacing the full path of the WHERE conditions by partial paths and wildcards (*). For example, the path $root/node1[@attribute = '1']/node2[@attribute = '2']/nodeN[@attribute = 'N']/value is replaced by:

$root//*[@*attribute = 'N'*]*/value*          (a), or

$*root//nodeN*[@*attribute = 'N']/value*  (b)

### Indexing

Each database has its own indexing mechanisms. In MySQL, each query uses a single table, and the indexes were created for the variables that compose each condition in the query. In BaseX, structural indexes are created by default and the user cannot change them. One may choose to activate other kind of indexes, but one cannot create indexes other than those made available by the database. eXistdb, besides the structural indexes, allows the user to customize indexes. We followed the documentation provided by the developers and specified range indexes that supposedly would improve the response times. Indexes were specified for the **value**, **null_flavour** and **code_string** elements, and for the **archetype_node_id** and **type** attributes. Berkeley offers several indexing strategies and we specified indexes for the same element and attribute values used in eXistdb. As stated before, and contrary to the previous databases, in which an index may be reused in any query that could benefit from it, in Couchbase each query generates an index when it is published for general use. Therefore, indexes in Couchbase are specific to a query, not being reusable in others.

### Database Assessment in a Centralized Environment

The 8 queries were submitted to all databases, using their corresponding Java API. All databases were installed in a single machine, an Intel^®^ Core^™^ i7-3770S CPU @ 3.10GHz × 8, with 8 GBytes of memory, running Ubuntu 12.04 LS, 64 bits. The queries were submitted in sequence both for a smaller and a larger time interval to the sus10k, sus42k and sus100k datasets in the XML databases, and to all datasets in MySQL and Couchbase. Each query was submitted 20 times to Couchbase, MySQL and BaseX, 15 times to eXistdb and 10 times to Berkeley. Based on a previous study that compared the XML databases [18], we expected that the response times in eXistdb and Berkeley DB XML would be slower than BaseX. Therefore, we reduced the number of times the queries were submitted to those databases in order to reduce the evaluation time. The combination of rewriting and indexing strategy with best performance is shown for each database in the results section.

### Database Assessment in a Distributed Environment

Couchbase was tested for 1-, 2-, 4-, 8- and 12-node (computer) cluster setups with the number of dataset copies configured as 1, 2, 2, 3 and 4, respectively. The tests were performed on computers with 2 GB Memory, AMD Athlon Dual Core Processor, 2.1 GHz, running Ubuntu 12.04 LS, 32 bits. The network speed was 10 Mbit/s. The sus4200k dataset was not evaluated in the 1-node and 2-node cluster setup, because the machines’ memory sizes are not recommended for the size of this dataset. The queries were submitted 20 times to all datasets and all cluster configurations, both for the shorter and larger time intervals. The indexing times for each query in each dataset in each cluster configuration were recorded.

### Ethics Statement

This work has been approved by the research ethical committee of the Hospital Universitário Pedro Ernesto—Rio de Janeiro, Brazil (CEP/HUPE–CAAe: 06785512.4.0000.5259). Given the diverse nature and size of the datasets and the fact that the data is retrospective, ability to give consent was unfeasible for all patients. For the purposes of this study the information about the patients was anonymized and de-identified prior to analysis, through the removal from the databases of all detailed identifying demographic data of patients, health professionals and organizations. For the SISCOLO database, the date of birth and the date of exam were modified by adding or subtracting a random number of days (in the intervals ±912 and ±100 respectively). In the hospitalization, outpatient and mortality database, the date of birth, admission date, discharge date, validity dates, issue date and death date were shifted a random number of days in the interval (0, 912) at the same time for the same patient. This was done in order to obtain an anonymous set of data without impacting the representativeness of the dataset from a technical point of view.

## Results

First we present the results for database sizes, response times, and Couchbase indexing times on a centralized environment. Then we show the results of response and indexing times for the distributed NoSQL solution.

### Tests on a Centralized Environment

#### Database Sizes

Table 1 shows the file and database sizes for each dataset when stored in a single machine. As expected, the JSON files are smaller than the XML files (around 35% smaller). Compared to the original relational database in MySQL, Couchbase demands from 2.8 to 5.8 times more space for the datasets sus10k to sus4200k, respectively. Of the XML databases, BaseX requires less space than the other two (eXistdb– 2.7 times more space and Berkeley– 3.2 times more space), but is much more space-consuming than Couchbase (3.8 times for sus10k to 5.8 times for sus100k).

**Table 1:** Size of Files and Databases (GB).

Storage Structure	DBMS	Datasets					
		sus10k	sus42k	sus100k	sus420k	sus1000k	sus4200k
**XML files**	-	3.2 x 10^−1^	1.4	3.2	1.4 x 10^1^	3.3 x 10^1^	1.4 x 10^2^
**JSON files**	-	1.9 x 10^−1^	8.3 x 10^−1^	2.0	8.3	2.0 x 10^1^	8.3 x 10^1^
**Relational DB**	**MySQL**	2.6 x 10^−2^	9.1 x 10^−2^	1.5 x 10^−1^	4.3 x 10^−1^	9.2 x 10^−1^	3.6
**XML DB**	**BaseX**	2.8 x 10^−1^	1.2	2.8	-	-	-
	**eXistdb**	7.6 x 10^−1^	3.3	7.6	-	-	-
	**Berkeley**	8.9 x 10^−1^	3.8	8.9	-	-	-
**JSON DB**	**Couchbase**	7.3 x 10^−2^	2.1 x 10^−1^	4.8 x 10^−1^	2.1	4.5	2.1 x 10^1^

#### Response Times

**Fig 2:**
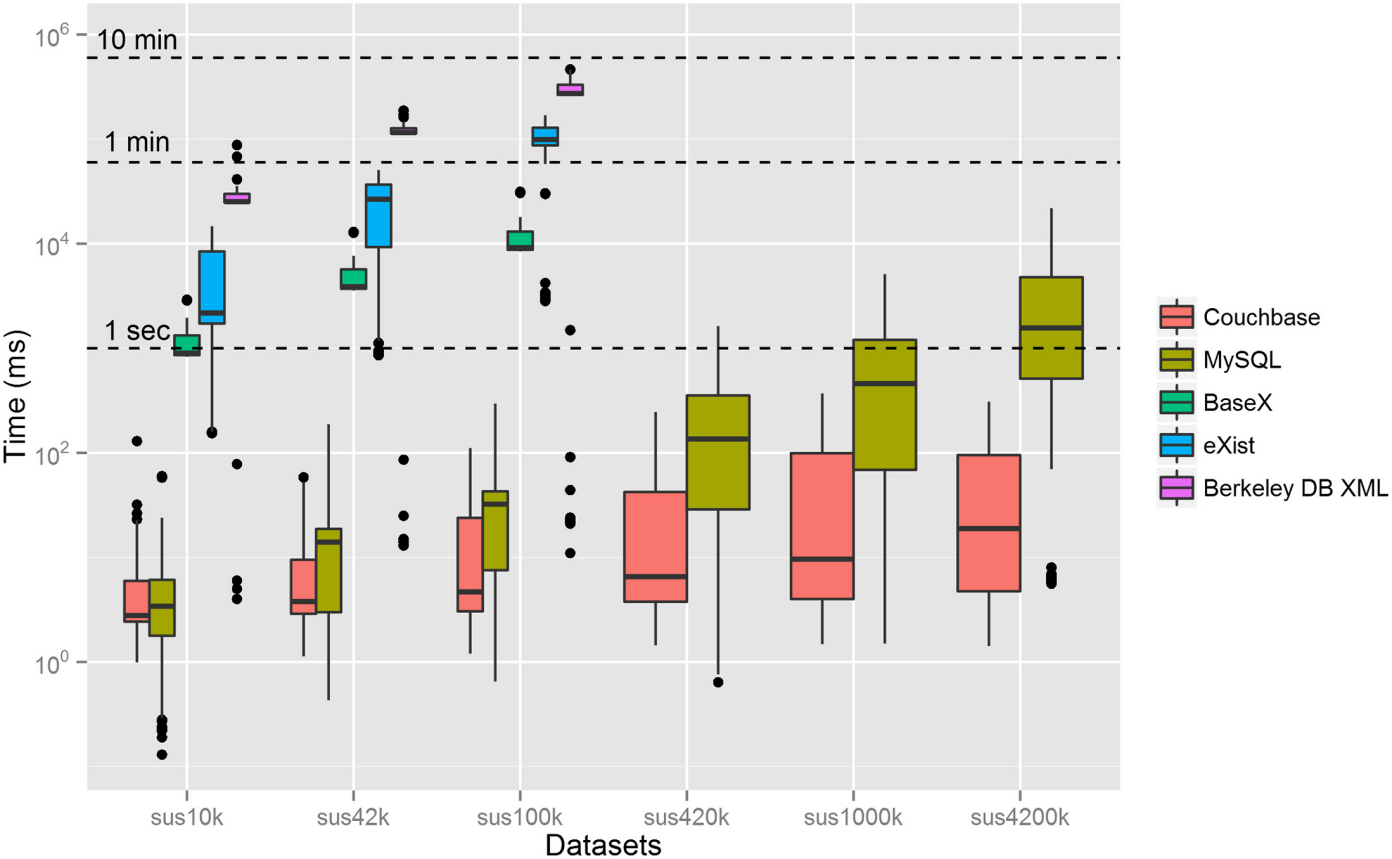
Boxplot of the response times for all queries grouped by databases and datasets. The dots represent outliers, which result usually from: *i)* faster response for some queries due to indexing in Berkeley and eXistdb, *ii)* and slower response for the first time each query is submitted to the databases. The y-axis is in the logarithmic scale. MySQL is the original relational database. BaseX, eXistdb and Berkeley DB are the XML databases and Couchbase is the JSON database.

Fig 2 shows the boxplot of the response times for all queries grouped by databases and datasets. In our tests, the main factors associated with the DBMS response time were the query types (or complexity), the dataset size and the result set size (associated with the query time span). The response times of the MySQL database were dependent only on the type of query and the size of the table, or dataset, to be searched. Similarly, for the XML databases the response times depend on the type of query and the size of the dataset and not so much on the time interval or the size of the result set. For Couchbase, the response times were neither dependent on the type of query nor on the size of the dataset. As it will be shown in the section on clustering results, the response times depended more on the size of the result set of the query.

Table 2 shows the descriptive statistics for the boxplots in Fig 2. In general, Couchbase has better performance than MySQL as the dataset size increases and so does the difference between the median values, which are respectively 1 order and 2 orders of magnitude for the sus1000k and sus4200k datasets. On average, Couchbase performs at least 2 orders of magnitude faster than the fastest tested XML databases (BaseX).

**Table 2:** Descriptive Statistics for the Boxplots of Fig 2.

Dataset	DBMS	Mean (ms)	SD^a^ (ms)	Min (ms)	P_25_^b^ (ms)	Median (ms)	P_75_^b^ (ms)	Max (ms)
**sus10k**	**Couchbase**	5.6	8.5	1.3	2.5	2.9	6.6	1.3 x 10^2^
	**Mysql**	4.7	5.9	1.3 x 10^−1^	1.8	3.4	6.1	6.0 x 10^1^
	**BaseX**	1.3 x 10^3^	6.6 x 10^2^	8.3 x 10^2^	8.6 x 10^2^	9.0 x 10^2^	1.3 x 10^3^	2.9 x 10^3^
	**eXistdb**	4.9 x 10^3^	4.0 x 10^3^	1.5 x 10^2^	1.7 x 10^3^	2.2 x 10^3^	8.4 x 10^3^	1.5 x 10^4^
	**Berkeley**	3.0 x 10^4^	2.1 x 10^4^	4.0	2.5 x 10^4^	2.5 x 10^4^	3.0 x 10^4^	8.8 x 10^4^
**sus42k**	**Couchbase**	1.1 x 10^1^	1.4 x 10^1^	1.1	2.8	3.8	9.7	5.8 x 10^1^
	**Mysql**	1.3 x 10^1^	1.7 x 10^1^	4.3 x 10^−1^	3.0	1.4 x 10^1^	1.9 x 10^1^	1.9 x 10^2^
	**BaseX**	5.5 x 10^3^	3.0 x 10^3^	3.6 x 10^3^	3.7 x 10^3^	3.9 x 10^3^	5.7 x 10^3^	1.3 x 10^4^
	**eXistdb**	2.5 x 10^4^	1.4 x 10^4^	8.6 x 10^2^	9.3 x 10^3^	2.7 x 10^4^	3.7 x 10^4^	5.1 x 10^4^
	**Berkeley**	1.1 x 10^5^	4.9 x 10^4^	1.3 x 10^1^	1.1 x 10^5^	1.2 x 10^5^	1.3 x 10^5^	1.9 x 10^5^
**sus100k**	**Couchbase**	1.8 x 10^1^	2.6 x 10^1^	1.2	3.1	4.7	2.4 x 10^1^	1.1 x 10^2^
	**Mysql**	3.0 x 10^1^	3.1 x 10^1^	6.5 x 10^−1.^	7.5	3.2 x 10^1^	4.3 x 10^1^	3.0 x 10^2^
	**BaseX**	1.3 x 10^4^	7.2 x 10^3^	8.4 x 10^3^	8.7 x 10^3^	9.2 x 10^3^	1.3 x 10^4^	3.1 x 10^4^
	**eXistdb**	9.7 x 10^4^	4.3 x 10^4^	2.8 x 10^3^	8.7 x 10^4^	9.9 x 10^4^	1.3 x 10^5^	1.7 x 10^5^
	**Berkeley**	2.7 x 10^5^	1.2 x 10^5^	1.1 x 10^1^	2.6 x 10^5^	2.7 x 10^5^	3.3 x 10^5^	4.7 x 10^5^
**sus420k**	**Couchbase**	4.4 x 10^1^	6.7 x 10^1^	1.5	3.9	7.1	4.6 x 10^1^	2.5 x 10^2^
	**Mysql**	1.8 x 10^2^	2.0 x 10^2^	6.4 x 10^−1^	2.9 x 10^1^	1.4 x 10^2^	3.5 x 10^2^	1.6 x 10^3^
**sus1000k**	**Couchbase**	5.7 x 10^1^	8.8 x 10^1^	1.8	4.2	9.9	1.0 x 10^2^	3.7 x 10^2^
	**Mysql**	8.0 x 10^2^	1.1 x 10^3^	1.5	6.9 x 10^1^	5.0 x 10^2^	1.2 x 10^3^	5.1 x 10^3^
**sus4200k**	**Couchbase**	6.0 x 10^1^	7,7 x 10^1^	1.8	4.8	2.1 x 10^1^	9.5 x 10^1^	3.1 x 10^2^
	**Mysql**	3.4 x 10^3^	5.0 x 10^3^	5.6	5.1 x 10^2^	1.6 x 10^3^	4.8 x 10^3^	2.2 x 10^4^

MySQL is the original relational database. BaseX, eXist and Berkeley DB are the XML databases and Couchbase is the JSON database.

^a^ SD stands for standard deviation.

^b^ P_25_ and P_75_ stands for the lower and upper quartile, respectively.

The response time varies greatly for the different DBMS, datasets and queries. In general, as expected, the smaller number of documents (or rows) in the dataset, the faster is the response time. The response time of MySQL varies from 0.13 ms for query 3 and sus10k dataset to 2.2 x 10^4^ ms for query 2 and sus4200k dataset. For the XML databases, Berkeley XML DB has the highest response time variance, achieving 4.0 ms for query 8 and sus10k dataset and 4.7 x 10^5^ ms for query 1 and sus100k dataset. eXistdb response time varies from 1.5 x 10^2^ ms for query 3 and sus10k dataset to 1.7 x 10^5^ ms for query 1 and sus100k dataset. BaseX response time varies from 8.3 x 10^2^ ms for query 3 and sus10k dataset to 3.1 x 10^4^ ms for query 8 and sus100k dataset. Finally, the response time of Couchbase varies from 1.1 ms for query 8 and sus10k dataset to 3.7 x 10^2^ ms for query 5 and sus1000k dataset.

**Fig 3:**
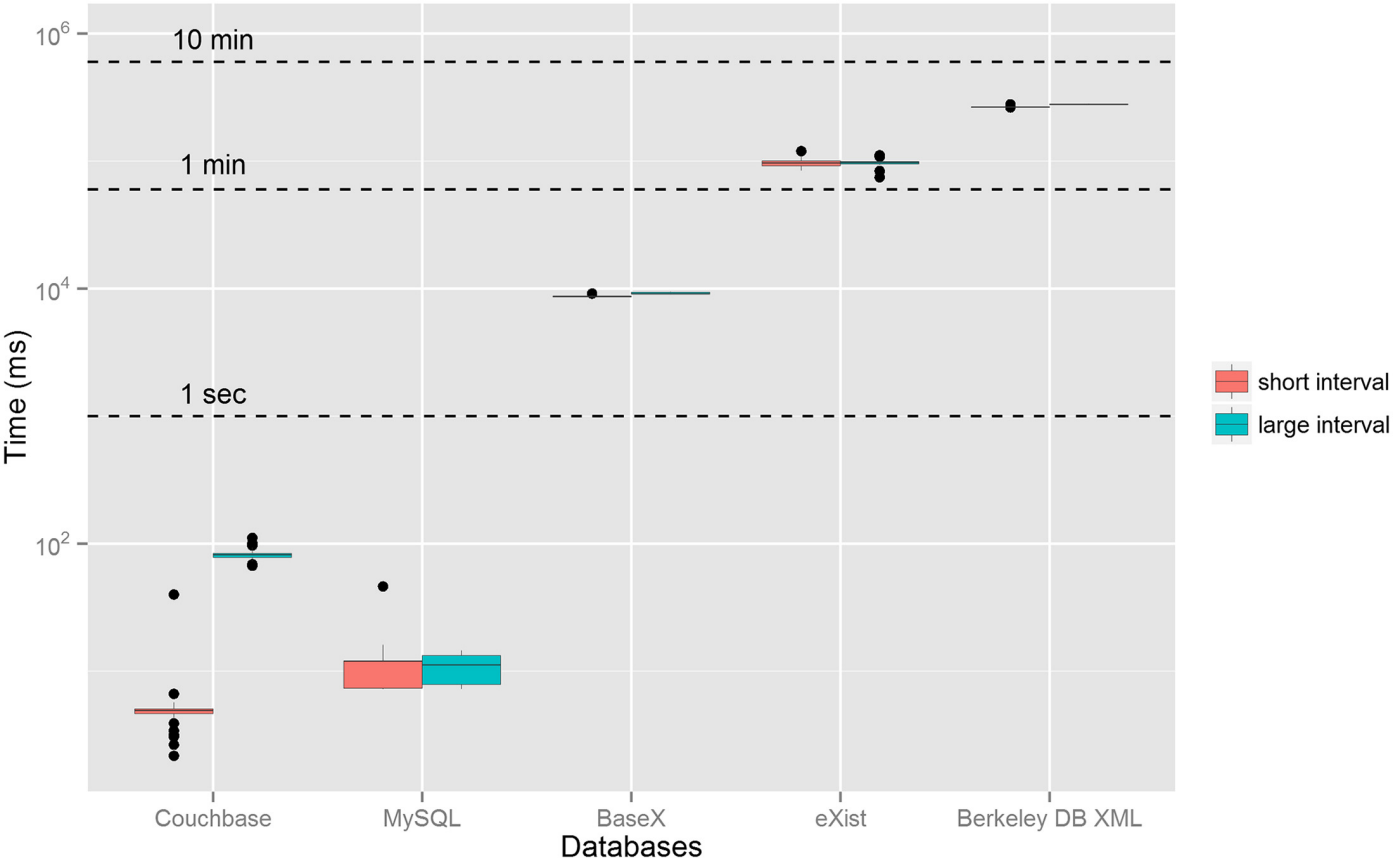
Boxplot of the response times for the sus100k dataset and one of the queries. The boxplots are grouped by database and date interval. The y-axis is in the logarithmic scale. The outliers above the median are due to the fact that usually the response time of the first submissions of the query to the database is higher than the others. The tests were performed on a single computer. MySQL is the original relational database. BaseX, eXistdb and Berkeley DB are the XML databases and Couchbase is the JSON database.

Fig 3 shows the boxplot of the response times for the sus100k dataset and query 4 grouped by database and date interval. The sizes of the result sets were 111 and 6,922 items for the short and large intervals, respectively. As shown in Fig 3, there is not much variability in the response times for query 4 in the XML databases and this pattern is similar for the other queries. As already discussed, the size of the result set has a significant impact on the response times in Couchbase, but it does not have a major influence on the other databases.

In the comparison amongst the XML databases, we notice that some databases might respond faster to some kinds of queries. However, for the specific queries used in this study, on average, BaseX outperforms eXistdb, which outperforms Berkeley DB XML. BaseX had better response times than eXistdb for all the queries, except for query 3, where the range index created in eXistdb improved its performance. The difference between their response times usually increased with the size of the dataset. On average, eXistdb had better response times than Berkeley DB XML, except for query 8 where the *edge index* for the "value" element generated faster response times for all three datasets, shown by dots in Fig 2. Despite being the slowest DBMS on average, Berkeley XML DB generated the fastest response times for query 8 amongst the three XML databases.

Query rewriting for the XML databases produced mixed results. For BaseX, the best response times for each query were obtained when the queries were rewritten according to the items 1 and 2.a in the section on query rewriting. On the other hand, for eXistdb, the rewriting of the queries according to the items 1 and 2.b produced the best response times for query 1 and sus10k dataset, query 4 and sus10k dataset, query 6 and sus10k dataset, and query 3. The original query produced the best results for query 8. For all other queries and datasets, the best response times for queries were obtained when the queries were rewritten according to the items 1 and 2.a in the section on query rewriting. Finally, for Berkeley XML DB, the rewriting of the queries according to the items 1 and 2.b produced the best response times for all queries.

#### Couchbase Indexing Times

**Fig 4:**
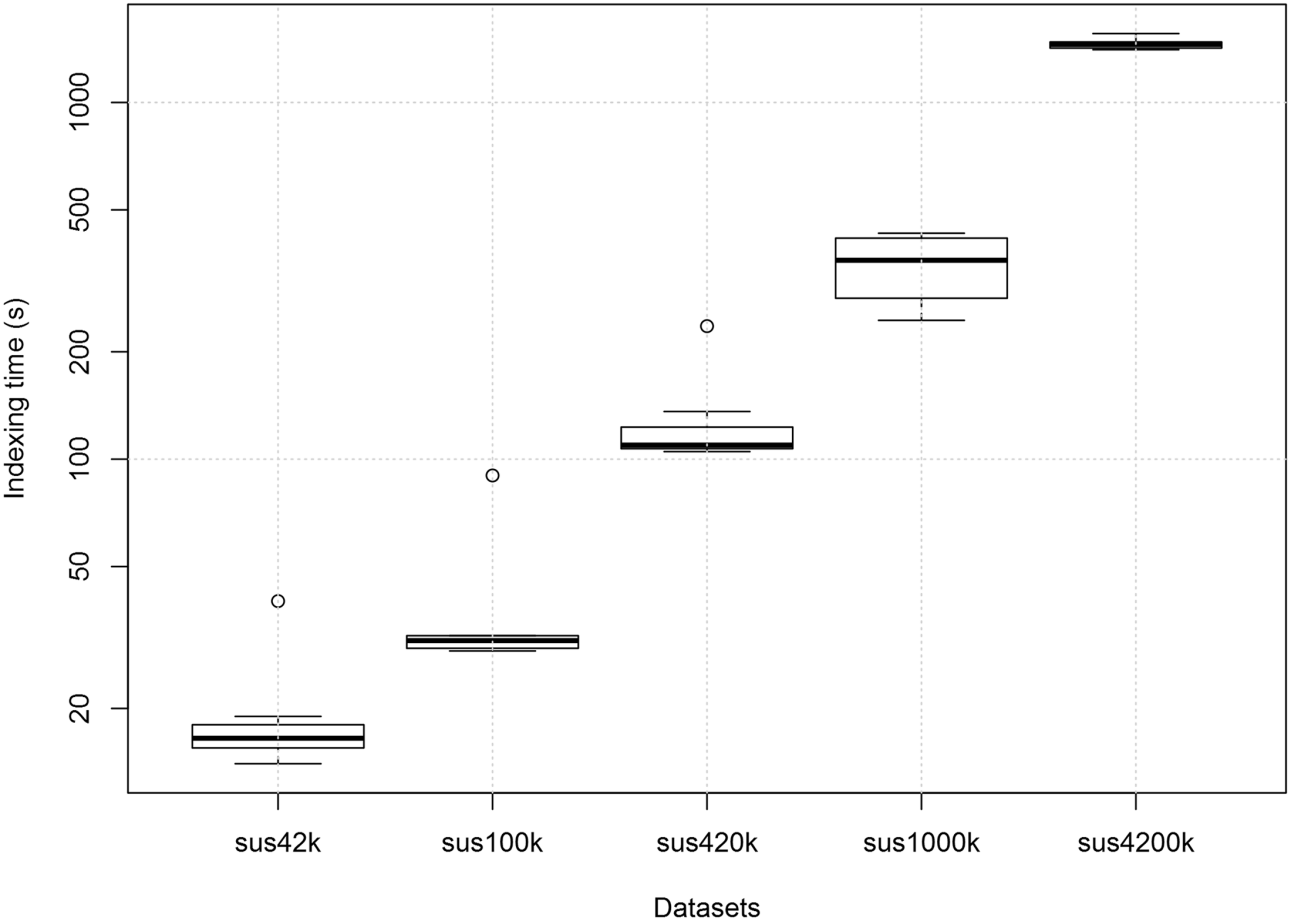
Boxplot of the indexing time for each dataset in Couchbase. Each boxplot contains 8 indexing measurements for each query. The outliers are due to the fact that usually the indexing time of the first query submitted to the database is higher than the others. The indexing times for the sus10k dataset were smaller than 1 s and were not shown. The measurements were performed on a single computer.

Fig 4 shows the boxplot of the indexing time for each dataset in Couchbase. It can be seen that, as expected, the indexing time increases significantly with the size of the dataset to be indexed. Moreover, the first time an index is created for a dataset, the indexing time is much larger, shown as outliers in the boxplot.

### Tests on a Distributed Environment

#### Indexing Times

**Fig 5:**
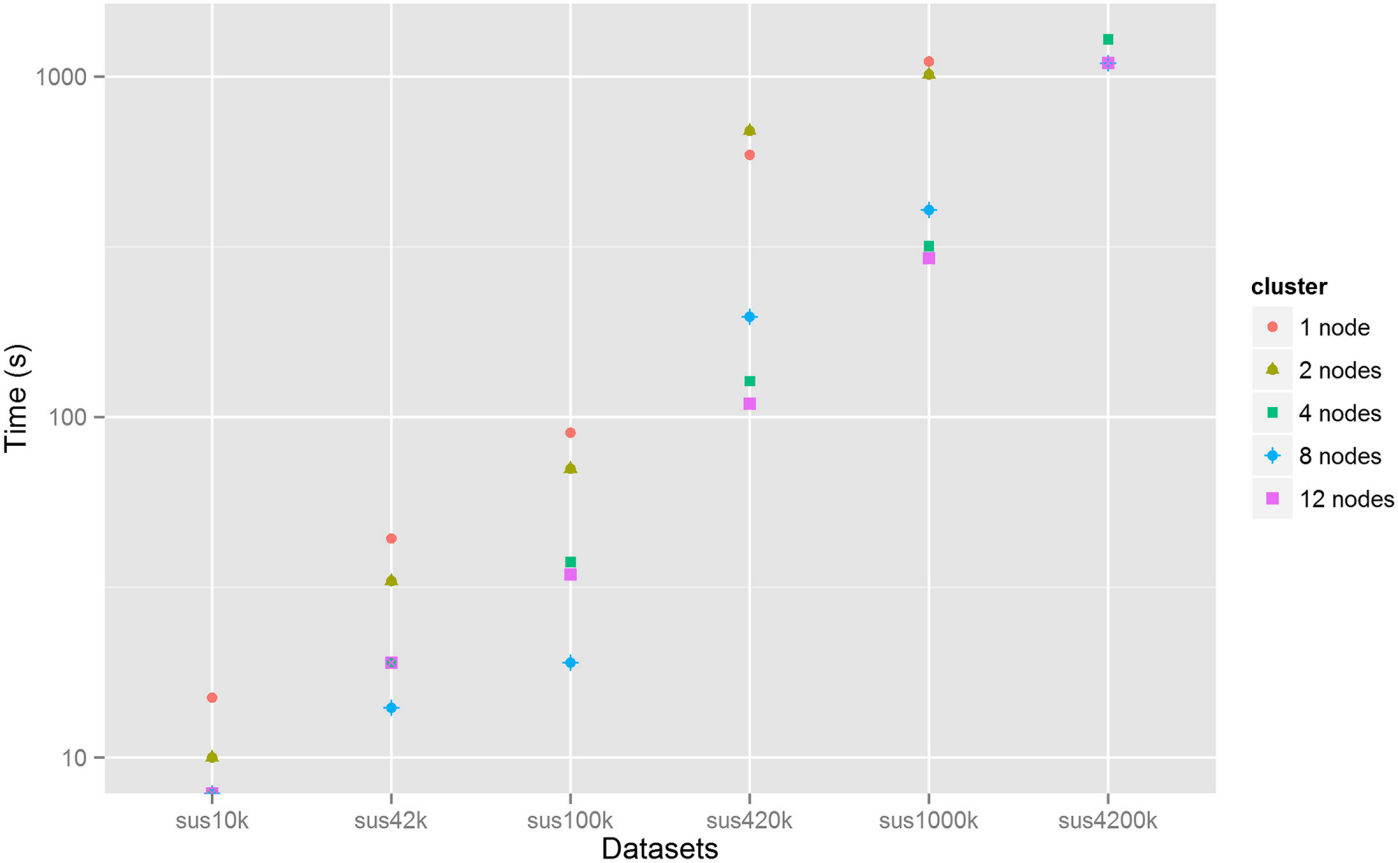
Median indexing time plotted versus dataset size for each cluster configuration. The y-axis uses a logarithmic scale. The indexing time was not measured in the 1-node cluster for the sus4200k dataset. In the 4-node, 8-node and 12-node clusters, the median indexing times for the sus10k dataset were rounded to 1 s and overlap in the graph.

All queries (views) submitted to Couchbase must first be indexed. After that, the database can be queried for the results. In each cluster, for each dataset, 8 measurements were made, one for each query. Fig 5 shows the median indexing time (in seconds) against the dataset size for each cluster. It can be seen that, in general, the indexing time increases with the size of the dataset. For the 12-node cluster, for instance, the median indexing time ranges from less than a second for the sus10k dataset to approximately 19 minutes for the sus4200k dataset. For each dataset, the indexing time usually decreases with the number of nodes. For the sus100k dataset, for instance, the median indexing time ranges from 90 s for the 1-node to 34.5 s for the 12-node cluster.

#### Response Times

**Fig 6:**
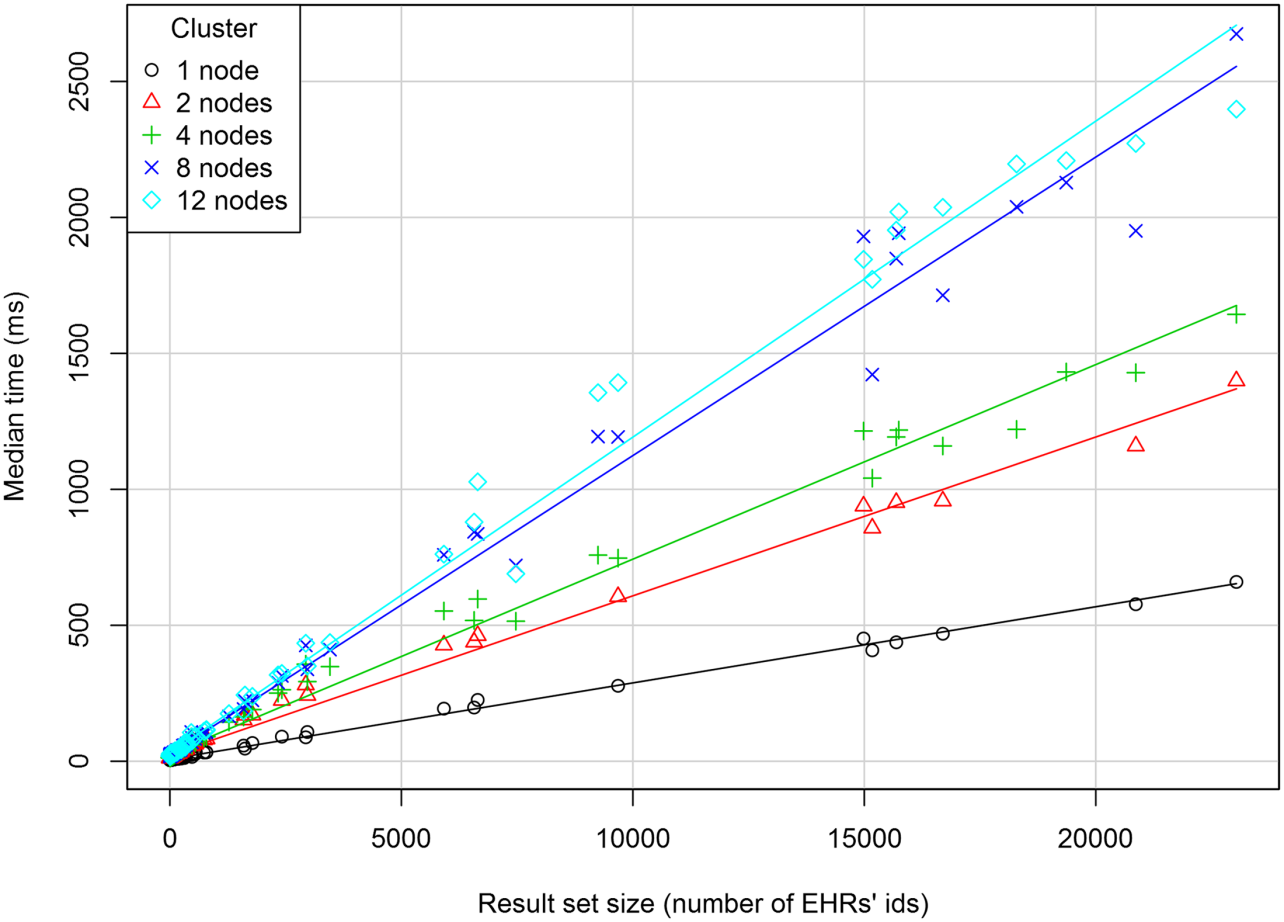
Scatterplot of the median response time against the result set size for each cluster. A regression line is plotted for each cluster.

Fig 6 shows the median response time (in milliseconds) against the size of the result set along with the regression line for each cluster configuration. The graph suggests a linear relationship between the two variables in each cluster. For the 1-node cluster, the median response time varies from 4 ms for a 1-sized result set to 6.6 x 10^2^ ms for the 23,036-sized result set. For result sets with the same size, the response times increase with the number of computers in the cluster. For the same cluster, the response time seems to depend on the size of the result set rather than on the size of the dataset.

A multiple linear regression model was adjusted to the relationship among the median response time as the dependent variable, the result set size as a quantitative independent variable and the cluster size as an independent factor. The statistics are: multiple R-squared = 0.86, adjusted R-squared = 0.86 and p-value < 2.2e^-16^. The model diagnostics shows some discrepancies with the assumptions of a linear regression model. However, as Fig 6 shows, a linear relationship between the size of the dataset and the median response time is a useful approximation.

## Discussion

In this study, we have evaluated the performance of several databases for managing openEHR models. The results confirm the limited performance of the assessed XML databases for population-based queries. Except for one query in Berkeley, the response times were still orders of magnitude slower than Couchbase, and the original relational DBMS. The on-line documentation of the XML databases was followed, changing the database configurations and setting indexes in order to enhance performance. Nevertheless, even with indexing and query rewriting optimizations, the evaluated XML databases are still far from the performance of standard, but inflexible, relational databases as previously suggested in [18, 19]. On the other hand, Couchbase results were very satisfactory, with response times in the order of a few seconds for databases with millions of patient records. The distributed nature of Couchbase seems to provide an advance in scalability compared to some of the previously published openEHR storage strategies.

If we think of the database schema in MySQL as a denormalized mapping of the archetypes used in this study, then Couchbase outperformed MySQL and would outperform the archetype-relational mapping proposal of Wang et al [15], as far as the query response times are concerned. Nevertheless, one must weigh this against the fact that Couchbase has to index each query and the indexing time increases with the size of the datasets. Furthermore, supposing that the relational model (MySQL) assessed in this study should outperform the corresponding generalized, more flexible, key-value pair table stored in relational databases, consequently, it is reasonable to suppose that Couchbase would also outperform the results of the generalized key-value pair table, as designed in [20]. Thus, Couchbase shows a good balance between performance and flexibility for storing openEHR data.

The queries used in this study were meant to be examples of epidemiological queries with different levels of complexity. Of course, they are not a representative sample of typical epidemiological queries, which is hard to achieve and would depart from the objective of this study. However, the variability and the patterns shown in the response times for each database (Fig 2) suggest that the queries were adequate to discriminate the performance of the databases, avoiding bias by choosing specific queries whose behaviour could be more homogenous and differ widely from the others.

An interesting result of the Couchbase cluster analysis is that the response time increases with the number of nodes. It seems that the gains that could be obtained from the data distribution among the nodes are outweighed by the time spent in the coordination of the nodes in the potentially too slow network. Larger cluster sizes may improve retrieval performances for much larger datasets or in clusters with faster machines and network speed and highly concurrent query accesses to the database. In the Kimura and Ishihara study [26], which used a cluster of MongoDB databases in a high speed network to store HL7 messages, performance increased up to 4 nodes in the cluster, after which it remained stable. Further studies of such concurrent updates and queries are needed.

There is very little data published about the performance of openEHR-based systems. This is in part due to the difficulty of creating a realistic test database with enough data to simulate real scenarios (different size hospitals, clinics, etc). In this study, patient data was anonymized from some Brazilian administrative public health information systems after integrating them by means of record linkage techniques. Then, all records belonging to the same patient were included in his/her EHR. The data contained in the administrative databases does not include the whole spectrum of real EHR records but includes the main types of variables present in EHRs, such as textual, temporal, coded and numeric variables. The EHRs are not very deep in the sense that the majority of records includes only one composition but they are not artificially generated data and the size of the database (~4 million records) makes it sufficient to challenge the performance of storage proposals.

While mapping the original relational database to openEHR Reference Model, some new data, such as data for auditability purposes, localization and version control, had to be included in the compositions due to the openEHR requirements. This new information plus the openEHR and XML verbosity caused the XML files to require much more space than the original relational data. However, they do not explain why the different XML databases differ widely in the space they use to store data, especially when this extra space does not correspond to better performance, measured by the response time of queries submitted to the databases. Since the JSON version of the XML files demands less space, Couchbase requires much less space than the XML databases.

It is unlikely (and even not necessary) to find a solution for storage systems that perfectly fits all purposes, that is, one that allows for single-patient queries, supports OLAP analysis and achieves reasonable response times for ad hoc queries. In traditional health information systems this leads to two types of models which are resource demanding to maintain when clinical needs change. For example, Couchbase enforces the BASE (Basically Available, Soft State, Eventually Consistent) philosophy that might cover many EHR use-cases, but it is subjected to the constraints of the CAP theorem which states that any networked shared-data system can have at most two of three desirable properties: consistency (C); high availability (A); and tolerance to network partitions (P) [43]. In a scenario where all the ACID (Atomicity, Consistency preservation, Isolation, Durability) [44] properties must be strictly enforced, we could have a mixed solution with both:

1. a traditional persistence mechanism, that has the ACID properties, which is used to deal with the individually focused writes and queries, and
2. another persistence mechanism which does not have the ACID properties, but provides good performance for population-based queries based on slightly delayed copies of data.

Maintaining two different persistence systems is likely to be a burden to the IT staff and adds to the cost of the system maintenance. There is thus a need for further research and clarification of use cases in archetype-based systems that might need a stronger consistency model than Couchbase provides.

The study of persistence mechanisms for archetype-based systems will remain an open issue. This study adds a drop to this ongoing debate and we encourage others to publish their results. The performance of Couchbase shows that it and other related NoSQL persistence mechanisms may be worth studying further, including the one suggested by [20] that uses the generalized key-value approach stored in a table in a SQL database, and other proposals that explore the MapReduce paradigm, such as MongoDB [25].

### Limitations of the Study

This study does not simulate a real production scenario with ad hoc population-based mixed with insertions, updates, queries for individual data, concurrent access, varying load and so on. It could be interpreted as a best response scenario, with only one user submitting a single query at a time.

In spite of the dataset used in this study being anonymous, there is no permission to make it public. It would be useful to have a mass of publicly available archetype-based EHR dataset that could be used by different groups to test the performance of persistence solutions. This is often commented upon in the openEHR discussion lists.

Our cluster analysis was limited by the speed of the network and the computational power of the nodes of the clusters. It would be interesting to reproduce the study on a faster network, with more powerful machines and also with larger datasets in order to have a better understanding of the effects that changing the number of nodes in the cluster have on the response times.

## Conclusions

This study investigated the performance on population-based queries of NoSQL persistence systems managing openEHR compliant data. The tested XML databases are on average much slower than both the original relational database (used as reference) and Couchbase when retrieving results from ad hoc population-based queries. Couchbase, in spite of requiring more space than the reference relational database and having a much larger indexing time for each new query, has better response times than the relational database. For scenarios where the ACID properties do not need to be strictly enforced, Couchbase seems to be a promising solution for retrieving results from population-based queries in systems persisting openEHR compliant documents.

## References

[1] Kimbal R. The data warehouse lifecycle toolkit. Indianapolis: Wiley Publishing, Inc; 2008.

[2] Immon WH. Building the data warehouse. 4th ed Indianapolis: Wiley Publishing, Inc; 2005.

[3] Johnson SB. Generic Data Modeling for Clinical Repositories. J Am Med Inform Assoc. 1996; 3(5):328–339. 8880680 10.1136/jamia.1996.97035024 PMC116317

[4] Nadkarni PM, Marenco L, Chen R, Skoufos E, Shepherd G, Miller P. Organization of Heterogeneous Scientific Data Using the EAV/CR Representation. J Am Med Inform Assoc. 1999; 6(6): 478–493. 10579606 10.1136/jamia.1999.0060478 PMC61391

[5] Beale T, Heard S. OpenEHR architecture overview, 2005. Available: http://www.openehr.org/releases/1.0.2/architecture/overview.pdf. Accessed 2016 Jan 15.

[6] International Organization for Standardization. IS 13606: Health informatics—Electronic healthcare record communication—Part 1: Reference Model. International Organization for Standardization, 2008. 83 p. Available: http://www.iso.org/iso/home/store/catalogue_tc/catalogue_detail.htm?csnumber=40784.

[7] The HL7 Version 3 Clinical Document Architecture (CDA^®^). Available: http://www.hl7.org/implement/standards/product_brief.cfm?product_id=7.

[8] Clinical Information Modeling Initiative (CIMI). Available: http://www.opencimi.org. Accessed 2016 Jan 15.

[9] Atalag K, Yang HY, Tempero E, Warren JR. Evaluation of software maintainability with openEHR—a comparison of architectures. Int J Med Inform. 2014; 83:849–859. 10.1016/j.ijmedinf.2014.07.006 25153769

[10] The openEHR-technical Archives. openEHR—Persistence of Data, 2012. Available: http://lists.openehr.org/pipermail/openehr-technical_lists.openehr.org/2012-February/006747.html. Accessed 2016 Jan 15.

[11] The openEHR-technical Archives. Dual Model EHR implementation, 2011. Available: http://lists.openehr.org/pipermail/openehr-technical_lists.openehr.org/2011-June/006068.html. Accessed 2016 Jan 15.

[12] Muñoz A, Solominos R, Pascual M, Fragua J, Gonzalez M, Monteagudo J, et al Proof-of-concept Design and Development of an EN13606-based Electronic Health Care Record Service. J Am Med Inform Assoc. 2008; 14(1):118–129. 10.1197/jamia.M2058 PMC2215080 17068357

[13] Frade S, Freire SM, Sundvall E, Patriarca-Almeida JH, Cruz-Correia R. Survey of openEHR storage implementations. Proceedings of the 26th IEEE International Symposium on Computer-Based Medical Systems 2013 June 20–22; Porto, Portugal. p. 303–307. Available: http://www.computer.org/csdl/proceedings/cbms/2013/9999/00/06627806.pdf.

[14] Beale T. Node + Path persistence. Available: https://openehr.atlassian.net/wiki/pages/viewpage.action?pageId=6553626. Accessed 15 January 2016.

[15] Wang L, Lingtong M, Wang R, Lu X, Duan H. Archetype relational mapping—a practical openEHR persistence solution. BMC Med Inform Decis Mak. 2015; 15:88 10.1186/s12911-015-0212-0 26541142 PMC4636072

[16] Standard Java libraries for Java implementations of openEHR. Available: https://github.com/openEHR/java-libs. Accessed 2016 Jan 15.

[17] Sundvall E, Nyström M, Karlsson D, Eneling M, Chen R, Örman H. Applying Representational State Transfer (REST) Architecture to Archetype-based Electronic Health Record Systems. BMC Med Inform Decis Mak. 2013; 13 10.1186/1472-6947-13-57 PMC3694512 23656624

[18] Freire SM, Sundvall E, Karlsson D, Lambrix P. Performance of XML Databases for Epidemiological Queries in Archetype-Based EHRs. In: Karlsson D, Elberg PB, Fossum M, Galster G, Hartvigsen G, Koch S, Nilsson G. Scandinavian Conference on Health Informatics. 2012 October 2–3; Linköping, Sweden. Linköping Electronic Conference Proceedings, 70, p 51–57. Available: http://www.ep.liu.se/ecp/070/009/ecp1270009.pdf.

[19] Green J. A Comparison of the Relative Performance of XML and SQL Databases in the Context of the Grid-SAFE Project. University of Edinburgh, 2008 Available: http://gridsafe.sourceforge.net/docs/database-benchmark-report.pdf.

[20] Lee KK-Y, Tang W-C, Choi K-S. Alternatives to relational database: Comparison of NoSQL and XML approaches for clinical data storage. Comput Meth Prog Bio. 2013; 110:99–109. 10.1016/j.cmpb.2012.10.018 23177219

[21] Marand Think!EHR Platform. Available: http://www.marand-think.com.

[22] The Apache Software Foundation, Apache Lucene. Available: https://lucene.apache.org/.

[23] Marco-Ruiz L, Moner D, Maldonado J, Kolstrup N, Bellika JG. Archetype-based data warehouse environment to enable the reuse of electronic health record data. Int J Med Inform. 2015; 84(9):702–714. 10.1016/j.ijmedinf.2015.05.016 26094821

[24] The Apache Software Foundation. Apache CouchDBTM. Available: http://couchdb.apache.org/.

[25] MongoDB Inc. mongoDB. Available: https://www.mongodb.org/.

[26] Kimura E, Ishihara k. Virtual file system on NoSQL for processing high volumes of HL7 messages. Stud. Health Technol Inform. 2015, 210: 687–691. 10.3233/978-1-61499-512-8-687 Available: http://person.hst.aau.dk/ska/MIE2015/Papers/SHTI210-0687.pdf. 25991240

[27] Parker Z, Poe S, Vrbsky SV. Comparing NoSQL MongoDB to an SQL DB. Proceedings of the 51st ACM Southeast Conference. Savannah, GA, USA. 2013, 6 pages.

[28] Freire SM, Almeida RT, Bastos EA, Cabral MDB, Souza RC, Silva MGP. A record linkage process of a cervical cancer screening database. Comput Meth Prog Bio. 2012; 108:90–101. 10.1016/j.cmpb.2012.01.007 22341207

[29] Freire SM, Souza RC, Almeida RT. Integrating Brazilian health information systems in order to support the building of data warehouses. Res Bio-Med Eng. 2014; 31(3): 196–207. 10.1590/2446-4740.0666

[30] Oracle Corporation. MySQL database. Available: http://www.mysql.com.

[31] Ocean Informatics. Ocean Archetype Editor. Available: http://www.openehr.org/downloads/archetypeeditor/home.

[32] Ocean Informatics. Ocean Template Designer. Available: http://www.openehr.org/downloads/modellingtools.

[33] Ocean Informatics. Clinical Knowledge Manager (CKM). Available: http://www.openehr.org/ckm/.

[34] Freemarker. Java Template Engine Library. Available: http://freemarker.sourceforge.net/.

[35] BaseX. The XML database. Available: http://basex.org/.

[36] eXistdb. Open Source Native XML Database. Available: http://exist-db.org/exist/apps/homepage/index.html.

[37] Oracle Corporation. Berkeley DB XML. Available: http://www.oracle.com/technetwork/products/berkeleydb/index-083851.html.

[38] Couchbase. Couchbase. Available: http://www.couchbase.com/.

[39] Dean J, Ghemawat S. MapReduce: Simplified data processing on large clusters. In Proceedings of Operating Systems Design and Implementation (OSDI). San Francisco, CA. 2004:137–150.

[40] Jaitla J. Couchbase 103: Q & A. 2013 Oct 29. Available: http://blog.couchbase.com/couchbase-103-q-and-a. Accessed 2016 Jan 15.

[41] Bastos EA. Estimativa da Efetividade do Programa de Rastreamento do Câncer do Colo do Útero no Estado do Rio de Janeiro. M. Sc. Dissertation. Rio de Janeiro: Universidade Federal do Rio de Janeiro. 2011. Available: http://objdig.ufrj.br/60/teses/coppe_m/EdianeDeAssisBastos.pdf. Portuguese.

[42] W3C. XQuery 1.0. Available: http://www.w3.org/TR/xquery/.

[43] Brewer E. CAP twelve years later: How the "rules" have changed”, IEEE Explore. 2012; 45(2):23–29.

[44] Haerder T, Reuter A. Principles of transaction-oriented database recovery. ACM Comput Surv. 1983; 15(4):287.

